# Rapid and Accurate Registration Method between Intraoperative 2D XA and Preoperative 3D CTA Images for Guidance of Percutaneous Coronary Intervention

**DOI:** 10.1155/2019/3253605

**Published:** 2019-08-22

**Authors:** Taeyong Park, Kyoyeong Koo, Juneseuk Shin, Jeongjin Lee, Kyung Won Kim

**Affiliations:** ^1^School of Computer Science and Engineering, Soongsil University, 369 Sangdo-ro, Dongjak-gu, Seoul 156-743, Republic of Korea; ^2^Asan Image Metrics, Clinical Trial Center, Asan Medical Center, University of Ulsan College of Medicine, 88 Olympic-ro 43-gil, Songpa-gu, Seoul 138-736, Republic of Korea; ^3^Department of Systems Management Engineering, Sungkyunkwan University, 2066, Seobu-ro, Jangan-gu, Suwon-si, Gyeong gi-do 440-746, Republic of Korea; ^4^Department of Radiology and Research Institute of Radiology, Asan Medical Center, University of Ulsan College of Medicine, 88 Olympic-ro 43-gil, Songpa-gu, Seoul 138-736, Republic of Korea

## Abstract

In this paper, we propose a rapid rigid registration method for the fusion visualization of intraoperative 2D X-ray angiogram (XA) and preoperative 3D computed tomography angiography (CTA) images. First, we perform the cardiac cycle alignment of a patient's 2D XA and 3D CTA images obtained from a different apparatus. Subsequently, we perform the initial registration through alignment of the registration space and optimal boundary box. Finally, the two images are registered where the distance between two vascular structures is minimized by using the local distance map, selective distance measure, and optimization of transformation function. To improve the accuracy and robustness of the registration process, the normalized importance value based on the anatomical information of the coronary arteries is utilized. The experimental results showed fast, robust, and accurate registration using 10 cases, each of the left coronary artery (LCA) and right coronary artery (RCA). Our method can be used as a computer-aided technology for percutaneous coronary intervention (PCI). Our method can be applied to the study of other types of vessels.

## 1. Introduction

Recently, cases of coronary artery disease have significantly increased owing to the extension of the average life expectancy and lack of exercise [1, 2]. Coronary artery disease is caused by the narrowing or closing of the coronary artery due to the stenosis and metabolic failure of a heart muscle [2]. A stent enthesis is used as a representative treatment method. In a stent enthesis, a metal epiploon is inserted and unfolded into a diseased artery to relieve stenosis [2, 3]. A stent enthesis is a noninvasive treatment method, which has advantages for a patient such as the minimization of physical and mental strain through a minimal incision, control, and anesthesia. [4]. However, as this treatment depends on 2D X-ray angiogram (XA) images and 3D vascular structures—which are understood primarily by medical doctors through intuition and haptic feedback—the accuracy of this difficult medical procedure is not guaranteed [5]. To compensate for these weaknesses, an assistance method to register and visualize both preoperative 3D computed tomography angiography (CTA) and 2D XA images in real time has been actively researched.

Extensive research has been performed on the 2D and 3D registration of coronary arteries. Kerrien et al. measured translational parameters between 2D and 3D images using normalized cross correlation (NCC) [6, 7]. Distortion coefficient, rotational angle, and scaling parameters were calculated using a calibration procedure. However, the method required extensive processing time for the exploration of the whole image during the optimization of NCC. Additionally, the 2D and 3D images were acquired from the same equipment. Hipwell et al. proposed a similarity measurement method using pattern intensity (PI), gradient correlation, and gradient difference for 2D and 3D registration with a best neighbor optimization algorithm [8]. Although this method was accurate, it required extensive processing time. Metz et al. generated digitally reconstructed radiograph (DRR) images from CTA images and registered DRR and XA images using the NCC similarity measure [9, 10]. To minimize the deformation difference of vessels due to the heartbeat, 4D CTA images taken in multiphase were exploited. However, 4D CTA images are not usually taken in the general procedure of an intervention because of the high exposure to radiation compared to 3D CTA images. Additionally, the initial positions of vessels were manually aligned. Again, the NCC optimization procedure using a multi-resolution gradient ascent optimization technique required extensive processing time. Benseghir et al. performed 2D and 3D registration by comparing the similarities between a curved segment which was divided at branching or terminal points [11]. This method used Frechet distance and iterative closest curve (ICC). This method considered the geometric similarity between curves of vessels instead of only points—as in ICP. However, the computational efficiency was significantly degraded when considering multiple curves. Furthermore, a local minimum may have been encountered as this method did not consider the global search. Kaila et al. proposed a Gaussian mixture model of 3D centerlines from biplane XA and CTA images and maximized a posterior probability for registration using coherence point drift (CPD) [12] and expectation-maximization (EM) [13]. The robustness of this method was increased by using vessel diameter and branch point information. However, it required extensive processing time for 3D space registration and could not be applied to the general, single XA image. Kim et al. extracted vascular centerlines from XA and CTA images and projected 3D vessels onto 2D vessels for registration [14, 15]. In this method, the distances between the projected 3D vessels and 2D vessels were minimized by iteratively performing rotation, translation, and scaling transformations. The accuracy was further improved by performing thin plate spline-robust point matching (TPS-RPM) [16], which considers the local deformation of vessels. However, in this method, 3D vascular deformation was not considered because registration was performed only after the centerlines of 3D CTA vessels were projected onto the 2D images. Therefore, the topology information of the 3D CTA vessels was not preserved during registration. Additionally, owing to the lack of depth information, the convergence of local minima caused registration errors. Park et al. [17] proposed an initial registration using the principal axis generation and alignment with the bounding box such as a chessboard. For the accurate and fast fine registration, the trilateration method and Powell's optimization were performed. However, there is a disadvantage that the alignment of the cardiac cycles between two images is not taken into account and should be adjusted manually. Additionally, the principal axis used in the initial registration and the trilateration method caused registration errors due to the difference of the 2D and 3D segmentation, noise, etc. in the process of generation.

To address these limitations, in this paper, we propose a fast and accurate registration method for intraoperative 2D XA and preoperative CTA images of the same patient. Our method consists of three steps. First, to minimize the differences between two images, alignment of the cardiac cycle is performed based on electrocardiography (ECG) information. Second, a simulation environment is built similar to the acquisition timing based on DICOM (digital imaging and communications in medicine) information, and the gross transformation mismatch is corrected by optimal cube registration, including vessels. As this initial registration aligns the global rotation, scaling, and translation parameters between two images, it does not require distance map generation for the entire image. As such, the region of the distance map that uses local distance propagation is minimized. Following initial registration, faster and more robust convergence to the optimal value is achieved as the search space is limited to near the optimal value. The subsequent fine registration employs the selective distance measurement as a similarity measure to find the optimal transformation parameters and to minimize the distance between vessels. During fine registration, the importance value according to the anatomical structure of vessels is defined and exploited, resulting in more robust registration which is not affected by the difference of 2D and 3D segmentation, noise, etc. The proposed method minimizes unnecessary operations and enables fast and accurate registration by optimizing the performance of each step. We use 20 clinical datasets to evaluate the performance of our method in comparison to the registration accuracy, speed, and robustness achieved by Kim et al. [15] and Park et al. [17].

The remainder of this paper is organized as follows.  Section 2 describes the proposed registration method of 2D and 3D images—this procedure consists of three processing steps.  Section 3 presents the experimental results, followed by concluding remarks and a discussion of future work in Section 4.

## 2. Registration Method between 3D and 2D Images

**Figure 1:**
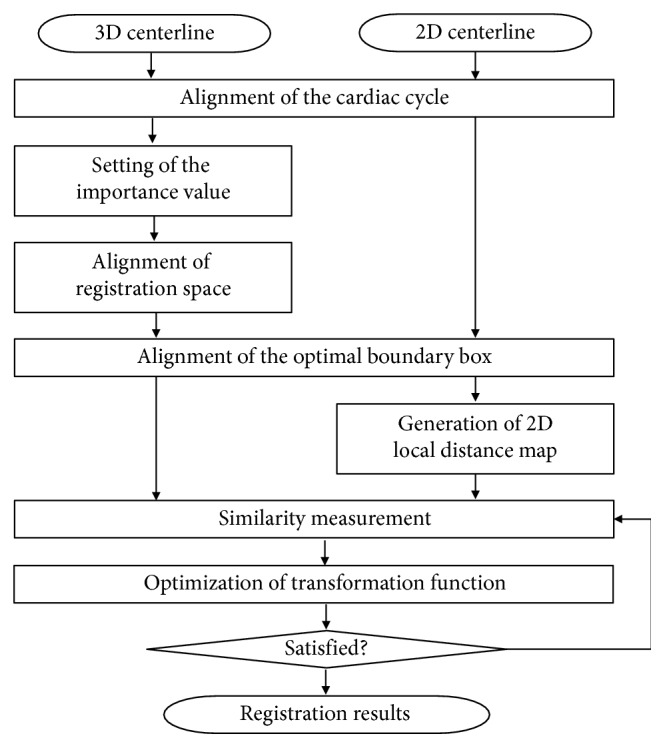
Registration of 3D CTA and 2D XA images.

The proposed method consists of the following steps, which are illustrated in Figure 1. For the registration of 3D CTA and 2D XA images of the same patient, each obtained from a different apparatus, cardiac cycle alignment and spatial alignment should first be performed. The ECG information of the 3D CTA and 2D XA images is used for this purpose. Subsequently, using DICOM information, we align the registration space by applying the environment while the 2D XA image is acquired into the 3D CTA image. However, as displacement information is missing from the DICOM information, an optimal boundary box is used to compensate for the lack. In order to perform fine registration, local distance propagation is performed to generate a 2D local distance map, and registration between the 3D CTA and 2D XA images is performed through an optimization process that uses selective distance measurement. In our method, the overall registration process uses a 3D centerline extracted from the 3D CTA image [18, 19] and 2D centerline extracted from the 2D XA image [20]. We input the normalized importance value into the 3D centerline and use it for registration. This feature reduces the probability of convergence to the local minimum, which allows for more robust convergence.

### 2.1. Matching of the Cardiac Cycle Using the ECG Information

**Figure 2:**
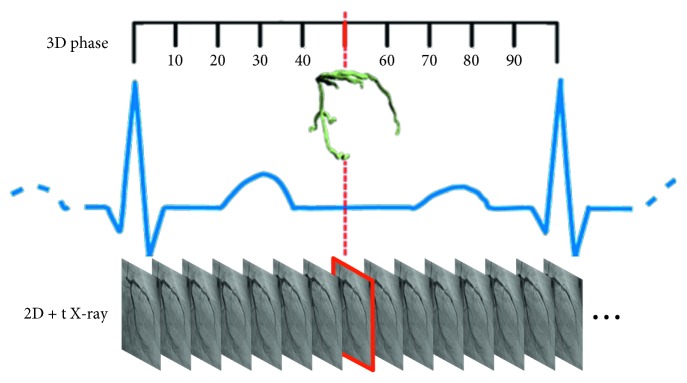
Matching process of the cardiac cycle using the ECG information.

The preoperative 3D CTA image may be acquired at any time during the cardiac cycle. Conversely, the intraoperative 2D XA images are acquired continuously. As such, a difference in vascular shape can occur between the images due to the different acquisition times in the registration process. Therefore, as shown in Figure 2, the process of matching the image acquisition times should first be performed.

In order to match the CTA and XA image acquisition times, an R-R interval is extracted from the ECG curve of the XA image and frames corresponding to the interval are distinguished. Subsequently, the cardiac cycle acquisition time of the CTA image is matched to the most similar frame of XA image. If it is matched with multiple frames, that by which the error is most minimized through comparison of the 3D and 2D centerlines is selected.

### 2.2. Setting of the Importance Value according to Anatomical Structure of the Vessel

**Figure 3:**
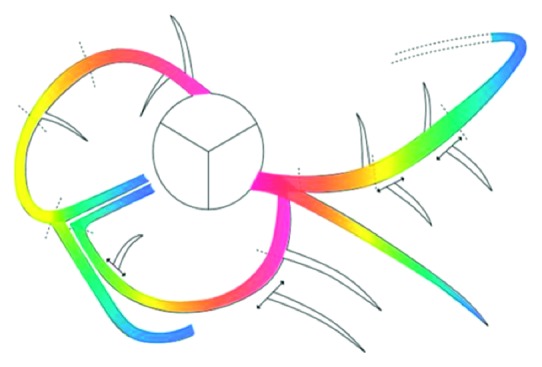
Representation of importance value according to the anatomical structure of the coronary arteries.

In this study, to improve the accuracy and robustness of registration, we input the normalized importance value based on the anatomical information of the coronary arteries into the 3D centerline extracted from the CTA image. We reflect the standardized anatomical information of coronary arteries, as recommended by the Society of Cardiovascular Computed Tomography (SCCT) [21], to ensure a general applicability. The importance value is applied by dividing the coronary arteries into main branch and sub-branch. The main branch is defined as RCA, LAD, and LCX based on the skeletonization information of Han et al. [18, 19]. Each main branch is divided into three areas of proximal, mid, and distal based on SCCT coronary segmentation information. Unlike the main branch, a sub-branch is not clearly distinguishable. Based on the main branch and SCCT coronary segmentation information, it is distinguished whether an unclear sub-branch exists mutually for each of the proximal, mid, and distal regions. If the number of sub-branches in each area is small or the same, it is considered to be an active sub-branch; otherwise, it is regarded as an inactive sub-branch. The importance value of the main branch is assigned to [1, 0.1] from the proximal to distal, as shown in Figure 3. The active sub-branch is gradually decreased from the bifurcation point of the main branch. The inactive sub-branch is assigned a value of 0.1.

The importance value of the 3D centerline enables the division of the main and sub-branches of the vessel. This feature reduces registration errors that converge to the local minimum because of low importance or unnecessary sub-branches. As such, it is possible to achieve robust registration.

### 2.3. Initial Registration between 2D and 3D Images

For multimodality image registration, alignment of the registration spaces should first be performed. For this purpose, we apply DICOM information obtained from the C-arm (the imaging apparatus of the 2D XA image) to the extracted centerline of the 3D CTA image. Subsequently, it is possible to quickly and accurately align the registration spaces of the 2D XA and 3D CTA images, using equation (1), for the initial registration:

(1) $$\begin{array}{c} \left[\begin{array}{c} x\\ y\\ 1 \end{array}\right]=K\left[\left.R\right|t\right]\left[\begin{array}{c} X\\ \begin{array}{c} Y\\ \begin{array}{c} Z\\ 1 \end{array} \end{array} \end{array}\right]=\;\left[\begin{array}{ccc} fm_{x} & 0 & p_{x}\\ 0 & fm_{y} & p_{y}\\ 0 & 0 & 1 \end{array}\right]\left[\begin{array}{cccc} r_{11} & r_{12} & r_{13} & t_{x}\\ r_{21} & r_{22} & r_{23} & t_{y}\\ r_{31} & r_{32} & r_{33} & t_{z} \end{array}\right]\left[\begin{array}{c} X\\ \begin{array}{c} Y\\ \begin{array}{c} Z\\ 1 \end{array} \end{array} \end{array}\right], \end{array}$$

where *x* and *y* are the coordinates of the 2D image, *X*,  *Y*,  and *Z* are the coordinates of the 3D image, *f* is the focal length, *m*_*x*_ and *m*_*y*_ are the pixels/mm, which are used when converting physical space into pixel space, *p*_*x*_ and *p*_*y*_ denote the principal points, and *r* and *t* denote the variables used for rotation and translation, respectively.  Table 1 shows the DICOM parameter information used in the initial registration.

**Figure 4:**
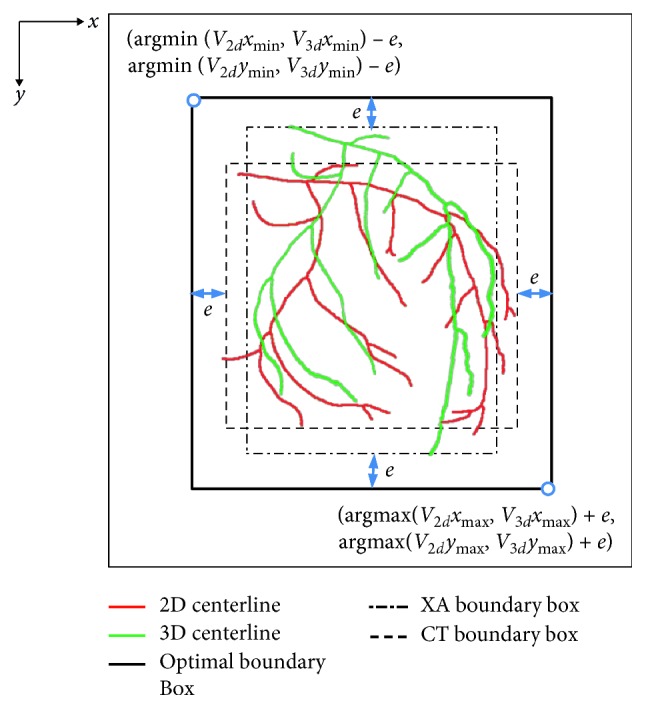
Generation of the optimal boundary box.

However, because the DICOM information obtained from the C-arm lacks translation variable information, it is necessary to acquire an estimated value. For this reason, we calculate the minimum and maximum values of the *x* and *y* axes of the projected 3D and 2D centerlines; then, we create a boundary box with (*x*_min_, *y*_min_) and (*x*_max_, *y*_max_) as the vertices, respectively. In addition, in order to consider a heartbeat and the geometric transformation in the registration process, we generate an optimal boundary box with added margin *e*, as shown in Figure 4. Subsequently, an initial estimate value of the translation variable is obtained by matching the optimal boundary boxes of the two images.

### 2.4. Generation of 2D Distance Map by Local Distance Propagation

In our method, as the global displacement between 2D and 3D centerlines is aligned during initial registration, a distance map for the entire area is unnecessary. To generate a 2D distance map, we consider eight-neighbor relations for distance propagation, as shown in equation (2). Let DP(*i*) be the propagated distance value of the pixel at the *i*-th position through the Euclidean distance, and the minimum distance value is allocated through comparison with the existing/conventional distance value. A distance *d*_max_ is assigned to the pixels whose value is not propagated:

(2) $$\begin{array}{c} \text{DP}\left(i\right)=\min\left(\underset{j\in 8-\text{neighbors}\left(i\right)}{\min}\text{DP}\left(j\right),\text{DP}\left(i\right)\right). \end{array}$$

**Figure 5:**
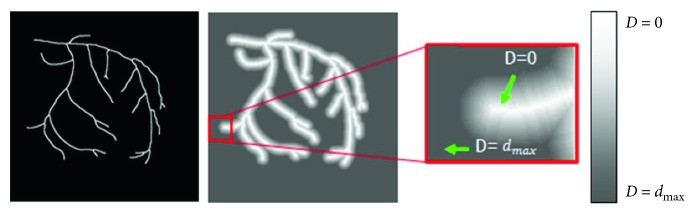
Generation of the 2D local distance map.

Figure 5 shows a 2D local distance map. The line displayed in white indicates the centerline extracted from the 2D XA image. The pixels having the same distance value from the centerline are displayed in the same color.

It is possible to measure one-step distance transformation, and it becomes unnecessary to calculate the distance of pixels with a distance value *d* > *d*_max_. Therefore, the processing time required to generate the distance map is minimized.

### 2.5. Similarity Measurement through Selective Distance Measurement

To identify similarities between the 3D and 2D centerlines, we use the distance value that is commonly used in the evaluation function of feature-based registration. The evaluation function has a minimum value when the 3D and 2D centerlines are aligned. For measurement of the evaluation function, we use the average of absolute distance difference (AADD), as shown in the following equation:

(3) $$\begin{array}{c} \text{AADD}=\frac{1}{N_{\text{C}}}\overset{N_{\text{C}}-1}{\underset{i=0}{\sum }}\alpha_{i}\left|2{\text{D}}_{\text{distanceMap}}\left(3p2{\text{D}}_{\text{centerline}}\left(i\right)\right)\right|, \end{array}$$

where *N*_C_ is the total number of 3D centerlines projected onto the 2D XA image, 3*p*2D_centerline_(*i*) denotes the *i*-th position of the 3D centerline projected onto the 2D XA image, *α*_*i*_ is the importance value of 3*p*2D_centerline_(*i*), and 2D_centerline_(*i*) denotes the 2D local distance map. In our method, the 3D centerline is superimposed upon the 2D local distance map [22], enabling the map to be utilized for the distance measurement between the two centerlines. As such, the processing time required to measure similarities between the centerlines is minimized.

### 2.6. Optimization of Transformation Function

Even if the same environment is applied at the XA image acquisition time using the DICOM information, initial registration errors can occur because of differences in protocols between imaging devices or other external factors. In order to reduce these errors, fine registration is subsequently performed using the transformation function of Powell's optimization method [23].

The transformation function consists of a translation vector (*T*_*x*_, *T*_*y*_, and *T*_*z*_) on the *x*, *y*, and *z* axis directions and a central rotation vector (*R*_*x*_, *R*_*y*_, and *R*_*z*_) on the *x*, *y*, and *z* axes, respectively. Optimization of transformation vectors simultaneously is inefficient with respect to both processing time and accuracy. In order to optimize the efficient transformation function, the translation-only *T*_T_ and rigid transformation *T*_R_ are sequentially performed, as shown in the following equation:

(4) $$\begin{array}{c} T_{T}\left(T\right)=\;M_{T}\left(T_{1}^{3}\right),\\ \;T_{R}\left(R\right)=\;M_{T}\left(R_{1}^{3}\right)\cdot M_{R}\left(R_{4}^{6}\right),\\ M_{T}\left(T\right)=\left[\begin{array}{cc} \begin{array}{cc} 1 & 0\\ 0 & 1 \end{array} & \begin{array}{cc} 0 & t_{x}\\ 0 & t_{y} \end{array}\\ \begin{array}{cc} 0 & 0\\ 0 & 0 \end{array} & \begin{array}{cc} 1 & t_{z}\\ 0 & 1 \end{array} \end{array}\right],\\ M_{R}\left(\theta \right)=\left[\begin{array}{cccc} c_{z}c_{y} & -s_{z}c_{x}+c_{z}s_{y}s_{z} & s_{z}s_{x}+c_{z}s_{y}c_{z} & 0\\ s_{z}c_{y} & c_{z}c_{x}+s_{z}s_{y}s_{z} & -c_{z}s_{x}+s_{z}s_{y}c_{z} & 0\\ -s_{y} & c_{y}s_{x} & c_{y}c_{x} & 0\\ 0 & 0 & 0 & 1 \end{array}\right], \end{array}$$

where *c*=cos(*θ*) and *s*=sin(*θ*). In this study, once the initial registration is performed, the rotation and translation vectors in the transformation function are restricted as shown in equation (5). Additionally, we apply Powell's optimization method [23] to converge to the optimal position. As such, it is possible to enable fast and accurate registration:

(5) $$\begin{array}{c} \left|T_{x}\right|\leq d_{\max},\;\\ \left|T_{y}\right|\leq d_{\max},\;\\ \left|T_{z}\right|\leq d_{\max},\\ \left|R_{x}\right|\leq \text{thR},\;\\ \left|R_{y}\right|\leq \text{thR},\;\\ \left|R_{z}\right|\leq \text{thR}, \end{array}$$

where thR and *d*_max_ represent the threshold of rotation and translation vectors, respectively. These values are experimentally determined.

## 3. Experimental Results

In this study, we implement the proposed algorithm in C++ on a PC using Visual Studio 2010, installed on a Windows7 64bit operating system. The experiments are performed with an Intel® Core™ i7 3.4 GHz CPU and 8 GB of main memory. We test our method on 10 XA LCA and 10 XA RCA images from 20 patients. The XA and CTA data are obtained using a Philips digital C-arm and Siemens SOMATOM definition flash. The image size of each XA and CTA image is 512 × 512 pixels. The acquisition rate of the XA image is 15 fps. Tables 2 and 3 contain specific information on the remaining 2D XA and 3D CTA images.

### 3.1. Evaluation of Registration Accuracy

**Figure 6:**
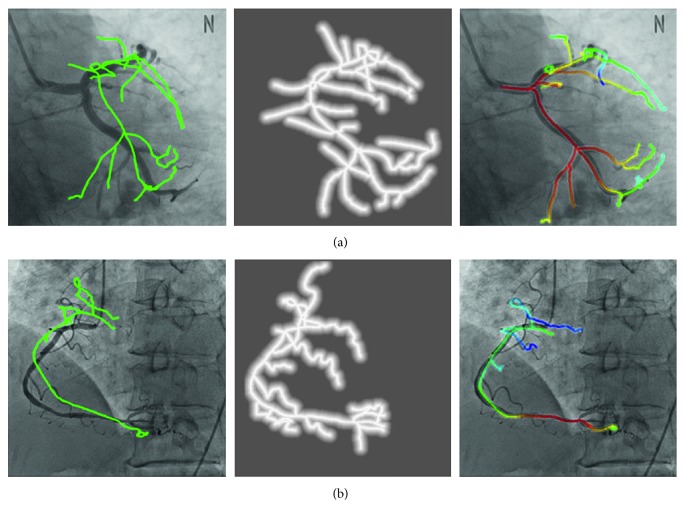
Registration process and results. Initial registration, distance map, and fine registration results of (a) LCA and (b) RCA.

The parameters for the rotation and translation vector threshold thR and *d*_max_ of the transformation function are 12° and 13 mm, respectively. The parameter is obtained by semiautomatic registration using the additional 2D-3D dataset, which is twice the mean of the measurements.  Figure 6 shows the result of the process applying the proposed method of the LCA of case 9 and RCA of case 18.

**Figure 7:**
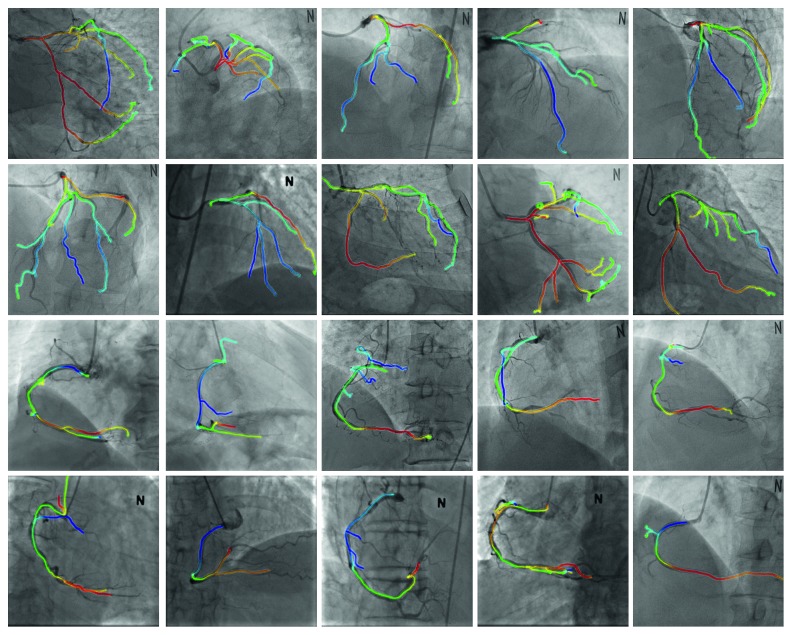
2D/3D registration results.

**Figure 8:**
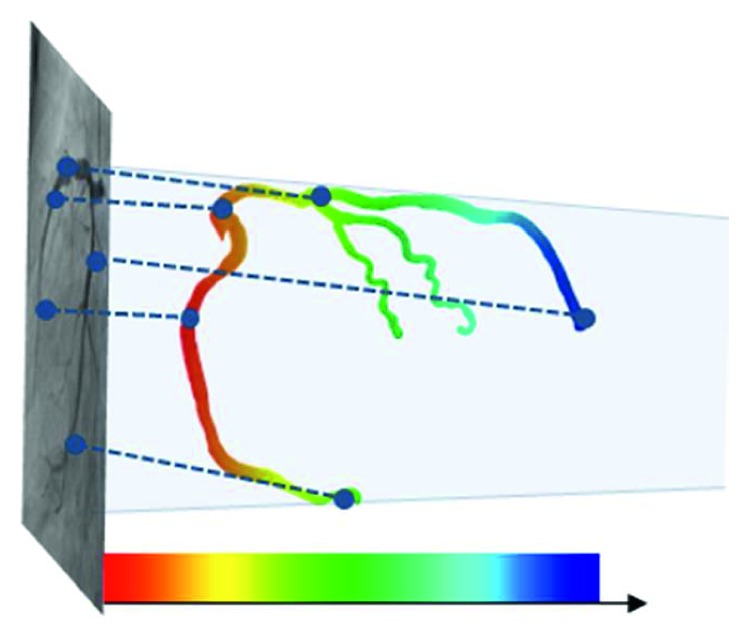
Centerline color representation according to the depth of the vessel.

Figure 7 shows the results of the proposed method for cases 1 through 20. In Figure 7, the first and second rows show the LCA results, whereas the third and fourth rows show the RCA results. The centerline color denotes depth information: shallow and deep vessel positions are indicated by blue and red, respectively, as shown in Figure 8. It is possible to visually confirm that the vessels of the preoperative 3D CTA and intraoperative 2D XA images are well-aligned, as shown in Figure 7.

To evaluate the accuracy of our algorithm, we compare the registration results of our method with the ground truth—manually annotated by the experts. The error of average difference is found to be minimal, even if it is aligned with an incorrect vessel. In this study, we use additional markers to measure the robustness of registration. The markers are manually input by the 3 experts on the 3D centerline and ground truth for the same corresponding point, respectively. The robustness of registration is measured using the average of distance difference (ADD) between markers, as shown in the following equation:

(6) $$\begin{array}{c} \text{ADD}=\frac{1}{M}\overset{M}{\underset{i=1}{\sum }}E\left(p_{i}-q_{i}\right), \end{array}$$

where *p* and *q* represent the markers of the 3D centerline and the ground truth, *E*(*p* − *q*) represents the Euclidean distance between *p* and *q*, and *M* represents the number of markers. 10 pairs are used for each patient.

Table 4 shows the accuracy and robustness results of the proposed method. It can be seen that performance related to the RCA is lower than that of LCA. This is because the variability of the RCA exceeds that of the LCA. As such, even if the same patient's data are used, the registration error of the RCA will be higher than that of the LCA.

### 3.2. Comparison with the Previous Method

**Figure 9:**
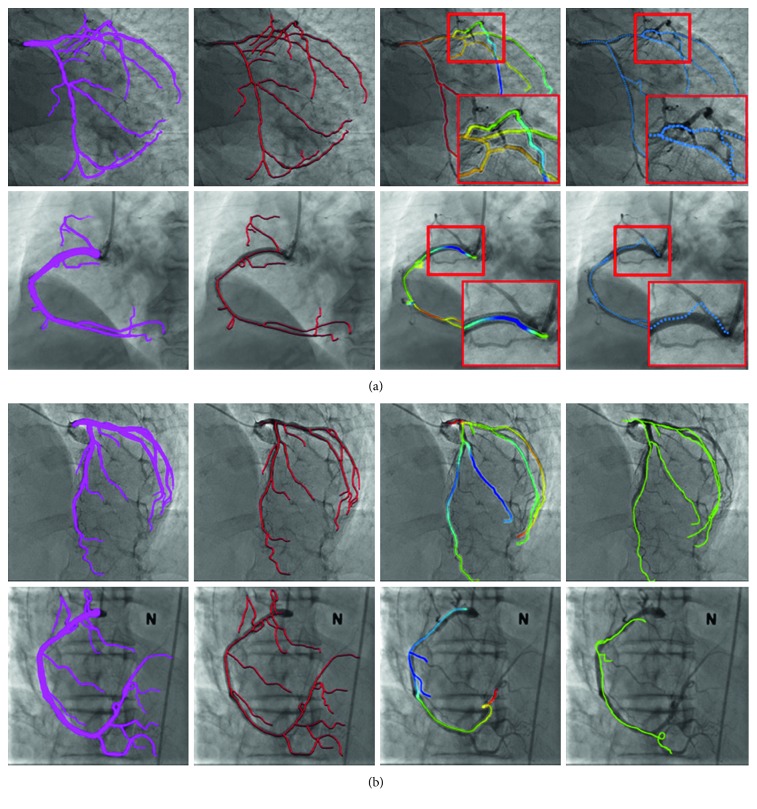
Registration results using the previous method and our method. (a) Ground truth (mask), ground truth (centerline), proposed method, and Kim et al. [15]. (b) Ground truth (mask), Ground truth (centerline), proposed method, and Park et al. [17].

In this study, we compare the registration accuracy and speed of our method with those of the methods suggested by Kim et al. [15] and Park et al. [17]. Experiments are performed on the same environment and data. For the accuracy evaluation, the ADD between the registration result and the ground truth is measured for the centerline, marker, and bifurcation points. The centerline accuracy results for the proposed method, Kim et al. [15], and Park et al. [17] are 1.2520 mm, 1.2237 mm, and 1.4098 mm, respectively. These values are observed to be numerically similar. However, as shown in Figure 9(a), the bifurcation points do not align in Kim et al. [15], where some vessels converge to a local minimum in a nonrigid registration process [15]. Additionally, the registration method proposed by Park et al. [17] shows the incorrect registration result due to the local error in the initial and correction registration process using the principal axis as shown in Figure 9(b).

**Figure 10:**
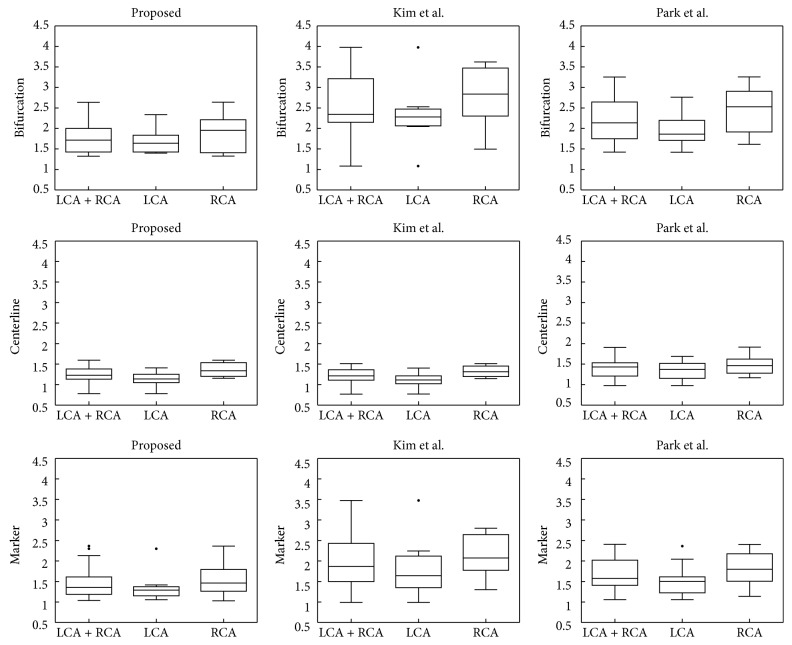
Average centerline, marker, and bifurcation error after registration using the previous methods and our method.

Figure 10 shows the accuracy and robustness results of the proposed method and previous methods. The proposed method is observed to be generally more accurate than the previous methods. In particular, accurately depicted vessel bifurcations are important for the precise insertion of surgical instruments and to understand the structure of the vessel during PCI. Therefore, if the accuracy is low, the vessel structure becomes difficult to analyze and understand.

**Figure 11:**
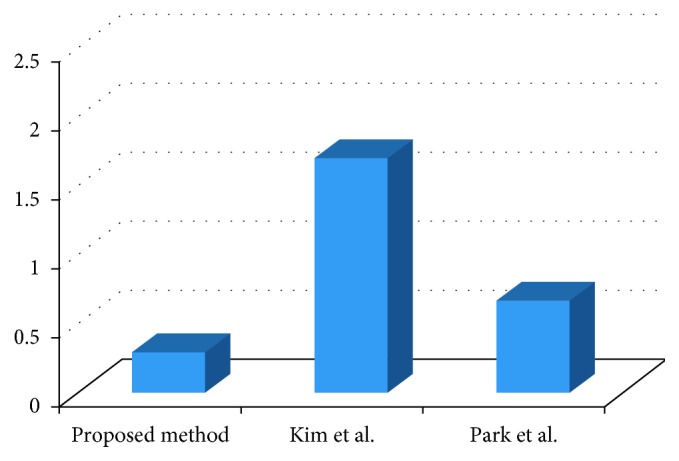
Average execution time of registration using the previous methods [15, 17] and our method.

Figure 11 shows the average execution times for registration using the proposed and previous methods. The average execution times of registration for the proposed method, Kim et al. [15], and Park et al. [17] are 0.2970 s, 1.7063 s, and 0.6712 s, respectively, in 20 clinical datasets. This demonstrates that the execution speed of the proposed method is faster than that of the previous methods. In this study, we perform fast and robust initial registration through the effective utilization of DICOM information. Additionally, we reduce the distance map generation time through local distance propagation. In the fine registration process, as initial registration has been performed, the search space for registration is restricted. As such, it is possible to further reduce the computation time. The proposed method minimizes unnecessary operations and enables fast and accurate registration by optimizing the performance of each step.

The registration method proposed by Kim et al. [15] attempts to improve accuracy through deformation of part of the vessel. However, it converges to the local minimum at the wrong place in a region with a local error, resulting in incorrect registration results. In particular, because a 3D centerline is projected once onto a 2D image and registration is performed only in 2D, the topology information of the 3D centerline extracted from a 3D CTA image cannot be preserved. In the registration method proposed by Park et al. [17], the overall registration process is influenced by the principal axis, thereby causing incorrect registration results for some dataset. Alternatively, in this study, we input the normalized importance value into the 3D centerline and use it for registration. This feature reduces the probability of convergence to the local minimum, which allows for more robust convergence. Furthermore, we can perform more accurate and robust registration between the 3D and 2D centerlines by preserving the topology information of the 3D centerline.

Table 5 shows the comparison of the previous methods with our method. The table shows that our number of test subjects exceed the other papers presented lately. Accuracy assessment metrics vary from paper to paper, and the target annotations (e.g., manual segmentation of vessels) also have a large variation so that it is very hard to find global criteria for comparison. However, we have tried to measure the accuracy, robustness, and clinical applicability of the registration by measuring the centerline, marker, and bifurcation of the vessel, respectively. As a result of the measurement, it can be confirmed that our method performed accurate, robust, and fast vessel registration with large test subjects. As such, our method can be used as a computer-aided technology for PCI.

## 4. Conclusion

To compensate for the limited 3D structure and depth information in 2D XA images, in this paper, we proposed a fast and accurate registration method for the fusion visualization of intraoperative 2D XA and preoperative 3D CTA images of the same patient. The proposed method consisted of three steps, namely, cardiac cycle alignment, initial registration, and fine registration.

First, to minimize the differences between two images according to the cardiac cycle, the cardiac cycles of the two images were aligned based on ECG information. Subsequently, a simulation environment was built similar to the acquisition timing, and the gross transformation mismatch was corrected by optimal cube registration, including vessels. An acquisition environment of 2D XA images in C-arm equipment was applied to 3D CTA images using DICOM information. As such, it was possible to quickly and accurately align the registration environment. An optimal boundary box was generated and aligned for the 3D and 2D vascular centerlines by rapidly compensating translational mismatch. As the initial registration aligned the global rotation, scaling, and translation parameters between the two images, this method lead subsequent registration to faster and more robust convergence to the optimal value. Additionally, the method did not require distance map generation for the whole image. As such, the region of the distance map using local distance propagation was minimized. During fine registration, the importance value according to the anatomical structure of vessels was defined and exploited, resulting in more robustness. The proposed method minimized unnecessary operations and enabled fast and accurate registration by optimizing the performance of each step. The experimental results showed registration errors of the whole vessel, anatomical landmarks, and branching points to be 1.252 mm, 1.458 mm, and 1.709 mm, respectively. The average processing time was 0.297 s. The proposed method performed fast and accurate registration between 2D XA and 3D CTA images, demonstrating the potential to provide doctors with substantial assistance during cardiac intervention. Future work will focus on a 2D + t/3D registration method by considering the correlation between 2D + t XA images.

## Figures and Tables

**Table 1:** DICOM parameter information.

Keyword	Tag
Positioner primary angle	(0018, 1511)
Positioner secondary angle	(0018, 1510)
Imager pixel spacing	(0018, 1164)
Distance source to detector	(0018, 1110)
Distance source to patient	(0018, 1111)

**Table 2:** LCA data for the XA and CTA images used in the experiment.

Case	XA					CTA	
	Positional primary angle	Positional secondary angle	Imager pixel spacing	Source to detector	Source to patient	Imager pixel spacing	Slice thickness
1	0	−38	0.244	990	649.23	0.358	0.5
2	57.5	−30	0.258	1208	765	0.335	0.75
3	47.5	17.2	0.258	1027	765	0.353	0.75
4	6.7	45	0.258	1202	765	0.304	0.75
5	35	29	0.293	990	668.152	0.390	0.5
6	41.35	16.93	0.258	−1000	765	0.339	0.75
7	−0.8	36.6	0.288	1070	720	0.332	0.5
8	−34	−30	0.293	1050	726.053	0.332	0.5
9	6.9	−43.5	0.258	1176	765	0.337	0.75
10	−33	−32	0.293	1120	744.444	0.390	0.5

**Table 3:** RCA data for the XA and CTA images used in the experiment.

Case	XA					CTA	
	Positional primary angle	Positional secondary angle	Imager pixel spacing	Source to detector	Source to patient	Imager pixel spacing	Slic thickness
11	−20.7	−20.6	0.287	1089	720	0.351	0.5
12	−35.6	1.7	0.287	990	720	0.351	0.5
13	−37.4	−0.5	0.287	1026	720	0.351	0.5
14	19.3	−24.2	0.287	1050	720	0.351	0.5
15	40.5	10	0.258	999	765	0.304	0.75
16	43.1	−0.38	0.259	1194	810	0.313	0.75
17	−33.1	0	0.259	1079	810	0.333	0.6
18	36	−3	0.293	1000	691.899	0.390	0.5
19	39.39	8.66	0.258	949	765	0.353	0.75
20	39.5	5.9	0.258	−1000	765	0.390	0.75

**Table 4:** Average centerline, marker, and bifurcation error after registration using the proposed method.

	LCA + RCA (mm)	LCA (mm)	RCA (mm)
Centerline	1.2520	1.1340	1.3700
Marker	1.4587	1.3528	1.5647
Bifurcation	1.8001	1.6853	1.9149

**Table 5:** Comparison of previous studies with our method.

Author	Modality	Dimensionality	No. of test subjects	Registration accuracy	Processing time
Kaila et al. [13]	Biplane XA/CTA	3D-3D	7	1.41 mm (RMSE^1^)	N/A
Khoo and Kapoor [24]	Biplane XA/CTA	2D-3D	6	3.8 mm (RMSD^2^)	0.4 s
				2.31 mm (RMSD^2^)	15 s
Liu et al. [25]	XA/CTA	2D-3D	10	0.6201pix (MPE^3^)	20 s
Park et al. [26]	XA sequence	2D-2D	9	7.02 mm (TRE^4^)	N/A
Our method	XA/CTA	2D-3D	20	1.252 mm (ADD^5^)	0.297 s

RMSD, root-mean-square distance; RMSE, root-mean-square error; MPE, mean projective error; TRE, target registration error; ADD, average of distance difference.

## Data Availability

They are clinical datasets, which cannot be opened publicly.
